# From Levin’s Universal Search to Policy-Guided Tree Search

**DOI:** 10.3390/e28040434

**Published:** 2026-04-13

**Authors:** Ming Li

**Affiliations:** School of Computer Science, University of Waterloo, Waterloo, ON N2L 3G1, Canada; mli@uwaterloo.ca

**Keywords:** Kolmogorov complexity, Levin’s universal search, reinforcement learning

## Abstract

Levin’s universal search embodies a striking principle: when a solution is efficiently verifiable, one can allocate search effort across candidate procedures according to a prior and obtain performance competitive (up to constant) with the best procedure in the reference class first published in (Levin, 1972). The accompanying video and Levin’s paper (Levin 2023) describe the history of this seminal result from the first-person perspective. While Andrey Kolmogorov passed away in 1987, Kolmogorov’s last student, Leonid Levin, systematically developed the theory of Kolmogorov complexity, including universal search. This perspective revisits the conceptual core of Levin-style universal search and finds its relationship to Solomonoff induction, a universal Bayesian framework for prediction that mixes over all computable hypotheses. Like Solomonoff induction, which serves as a spiritual foundation of large language models (LLMs), the Levin universal search has found important applications in AI. In this paper, we will follow one thread of this research on deterministic planning and reinforcement learning via policy-guided tree search.

## 1. Introduction

How do humans succeed in tasks that appear to require astronomical levels of search, such as proving deep theorems or predicting new particles? Levin [1,2] frames aspects of this question through inverting feasibly computable functions, optimal discovery algorithms, and the constant overheads that govern their practical performance [2]. In theoretical computer science, these themes naturally connect to *universal search*: instead of committing to a single algorithm for an unknown instance distribution, one should run a meta-procedure that allocates computation across candidate solvers, biased by a prior over descriptions.

The classical story is not merely “run everything in parallel.” The deeper claim is that there exist *optimal* or *bias-optimal* meta-procedures; for each instance, these perform within a multiplicative factor of the runtime of the best solver in the reference class, where the factor depends on the solver’s prior weight and on the universal procedure’s overhead. These overheads are precisely where theory meets practice. However, a guarantee can be simultaneously profound and unusable unless the search space, prior, and verification pipeline are engineered to keep constants under control.

In the last decade, a line of work has revived these ideas in a narrower but practically consequential setting: deterministic single-agent search and planning guided by a policy, with guarantees that resemble Levin-style bounds, although Levin universal search is not restricted to deterministic algorithms. In this setting, the “candidate procedures” become action sequences (or later, subsearches rooted at informative states), the prior becomes a policy over actions, and verification becomes simulation plus goal test. The result is a family of algorithms that inherit the universality template while making concrete design choices that reduce overhead and enable learning.

This study first provides a compact account of Levin-style universal search and why constant overheads are central to its practical importance. Second, it clarifies the relationship to Solomonoff induction: the two share a program-mixture structure but pursue different objectives, prediction versus solution finding. Third, it surveys three recent policy-guided search contributions and explains how each tightens the bridge from universal search theory to usable algorithms: LevinTS/LubyTS [3,4], LTS with convexly learnable context-model policies [5] (with a reproducible implementation [6]), and rerooted $\sqrt{\mathrm{LTS}}$ with decomposition-based competitiveness [7].

## 2. Universal Search: Core Idea and the Role of Overheads

### 2.1. The Meta-Principle: Allocate Compute by a Prior

At a high level, universal search operates over a family of candidate procedures—programs, proof attempts, heuristics, or other algorithmic descriptions. Each candidate *i* is assigned a prior weight $w_{i}\geq 0$ with $\sum_{i}w_{i}\leq 1$. A universal scheduler then distributes computation so that after a total time *T*, candidate *i* has received on the order of $w_{i}T$ steps (up to discretization and bookkeeping costs). Consequently, if some candidate $i^{★}$ solves the instance in $\tau_{i^{★}}$ steps, the universal procedure solves it in a time of the order of $\tau_{i^{★}}/w_{i^{★}}$, plus an overhead required to manage scheduling, execution, and verification.

This is the backbone of Levin-style optimality claims: the universal method is competitive with the best candidate in the reference class, paying an inverse-prior penalty and constant overhead. A useful mental model is that “universality” is obtained by refusing to bet on a single solver, while “optimality” is obtained by betting proportionally and ensuring that no solver is starved.

### 2.2. Inversion Problems and Verifiability

Universal search is most compelling when solutions are efficiently verifiable. A canonical scenario is inversion: given a feasibly computable function *f*, find a preimage *x* such that f(x)=y. Checking a candidate *x* requires computing f(x) and comparing it to *y*, which is feasible by assumption. In such settings, a universal search procedure is a generic inverting mechanism the performance of which is competitive with that of the best inverting strategy consistent with the chosen representation and prior, again up to overheads [2].

Deterministic planning offers a close analog. Here, the “witness” is an action sequence. Verification is simulation of the transition function followed by a goal test. This analogy is not only conceptual: it can be made algorithmic by defining a probability distribution over action sequences and then searching in increasing order of a cost that trades depth against probability mass.

### 2.3. Time-Bounded Complexity and Why Constants Matter

Levin’s ideas connect naturally to time-bounded Kolmogorov complexity, where description length and computation time are treated as resources. The operational viewpoint is that a hypothesis is not “simple” if it is short but takes infeasible time to run. Universal search embodies the same principle: it pairs a prior that favors short descriptions with a schedule that penalizes slow candidates.

## 3. Relationship to Solomonoff Induction

### 3.1. Solomonoff Induction in Brief

Solomonoff induction is a universal Bayesian framework for sequence prediction. It assigns prior weight to programs (commonly proportional to $2^{-|p|}$) and defines a mixture distribution by summing the weights of programs that output sequences consistent with observed data. Universality is expressed as dominance: the Solomonoff mixture multiplicatively dominates any computable measure up to a constant determined by the reference machine. Standard references include Solomonoff’s original work and modern expositions [8,9,10,11].

### 3.2. Shared Structure, Different Objective

Universal search and Solomonoff induction share a program-mixture structure and a competitiveness claim relative to a reference class. In Solomonoff induction, the mixture competes with the best computable predictor up to a multiplicative constant. In Levin-style universal search, although Levin was influenced by Kolmogorov [12] rather than Solomonoff, the scheduler competes with the best solver (program) up to an inverse-prior penalty and constant overhead. The objectives differ: Solomonoff induction seeks accurate prediction, whereas universal search seeks discovery of a witness that satisfies a verifiable relation. Levin (personal communication) points out: but there is another crucial relation for Solomonoff’s extrapolations. That extrapolations are based on finding short fast programs, which seems a hard NP problem (though not known to be complete). So optimal search is crucial for that (unless one-way functions do not exist). More specifically, this is an issue of one-way functions, rather than just worst-case P = ?NP problem. This subtlety is discussed in [13].

The bridge becomes explicit in universal-agent constructions such as AIXI, which combine a Solomonoff-style mixture over environments with planning over action sequences [11]. In such models, exact computation is infeasible, and resource-bounded Levin-style search is a natural ingredient in computable approximations. This is conceptually consistent with Levin’s emphasis on optimal discovery under feasible computation and the practical role of overheads [2].

### 3.3. Compression-Inspired Policies as a Partial Bridge

A practical bridge between Solomonoff-style prediction and Levin-style search is to use compression or prediction models as policies. In this view, a model that predicts likely next actions or transitions becomes a prior over trajectories, and search converts that prior into a correctness guarantee by enumerating or sampling trajectories in a principled way. This bridge is most explicit in the context-model formulation of LTS policies, which imports models from online compression and combines them via product-of-experts mixing [5]. The result is not Solomonoff induction itself, but it is strongly in the spirit of “prediction models become priors for search.”

## 4. From Universal Search to Policy-Guided Tree Search

A productive specialization of universal search replaces programs by trajectories, and priors over programs by policies over actions. The policy induces a distribution over action sequences, and search is organized to be fast when the policy assigns non-negligible mass to some solution trajectory. This section reviews three contributions that progressively enrich this specialization: first by establishing core enumeration and sampling guarantees, then by turning the bound into a learnable objective with convex structure, and finally by extending the universality template to decompositions via rerooting.

### 4.1. *LevinTS* and *LubyTS*: Enumeration and Sampling Guarantees

Orseau et al. introduce two policy-guided tree search algorithms for deterministic single-agent problems [3]. Nodes correspond to finite action sequences; the policy π assigns probabilities to actions and thus to sequences; and the goal is to reach any goal node. Their contribution is to show that policy guidance can yield guarantees on the number of node expansions that explicitly depend on the policy probabilities.

The first algorithm, LevinTS, is a best-first enumeration that expands nodes in increasing order of the cost

$$\mathrm{cost}\left(n\right)\;=\;\frac{d_{0}\left(n\right)}{\pi (n)},$$

where $d_{0}\left(n\right)$ is the sequence length and π(n) is the path probability under the policy. This cost mirrors the Levin trade-off: deeper and lower-probability candidates are delayed, but not ignored. The key theorem states that the number of expansions before reaching a goal is bounded by the minimum of this quantity over goal nodes:

$$N\left(\text{LevinTS},N^{g}\right)\;\leq \;\min_{n\in N^{g}}\frac{d_{0}\left(n\right)}{\pi (n)}.$$

The bound is instance-specific and policy-dependent. In particular, if there exists any goal-reaching trajectory that is relatively short and has non-negligible probability, then LevinTS is provably efficient.

A notable practical mechanism is *state cuts* for Markov policies. When the policy depends only on state, LevinTS prunes nodes that reach a state already visited with higher or equal probability. This converts the tree search into a form of graph search while preserving the intended best-first behavior in their analysis [3].

The second algorithm, LevinTS, uses sampling of trajectories and a restart schedule inspired by universal Las Vegas restarting strategies. While LevinTS is naturally suited to “needle-in-a-haystack” settings where few solutions exist, sampling can be dramatically faster when many solutions exist and the cumulative probability of solutions within a certain depth is large. Orseau et al. provide an expected expansion bound of the form

$$E\left[N\left(\text{LubyTS},N^{g}\right)\right]\;\leq \;\min_{d\in N_{\geq 1}}\;d\;+\;\frac{d}{\pi_{d}^{+}}\left(\log_{2}\frac{d}{\pi_{d}^{+}}+6.1\right),\;\pi_{d}^{+}=\sum_{n\in N^{g},\;d_{0}\left(n\right)\leq d}\pi \left(n\right),$$

which makes explicit how success depends on cumulative goal probability and the cost of not knowing the appropriate depth budget in advance [3,4].

Finally, the paper discusses how to avoid a brittle failure mode in which the policy assigns near-zero probability to the true solution path. Mixing the policy with a uniform component ensures exploration and yields modified guarantees that compare to the best among multiple policies up to a constant factor [3]. This can be read as an overhead-control mechanism: the algorithm pays a controlled multiplicative penalty to ensure that the search remains complete under a miscalibrated guide.

### 4.2. LTS with Context Models: The Bound Becomes a Convex Learning Objective

Orseau, Hutter, and Lelis revisit LTS from a learning-first viewpoint [5]. Their starting point is that the classical LTS expansion bound can be treated as a loss function. If a solution node *n* has depth d(n) and path probability π(n) under the policy, then d(n)/π(n) upper-bounds the number of expansions required to reach it (up to small additive constants depending on the exact definition). For a set of solved instances represented by solution nodes $N^{\prime }$, they define an LTS loss

$$L_{\mathrm{LTS}}\left(N^{\prime }\right)\;=\;\sum_{n\in N^{\prime }}\frac{d(n)}{\pi (n)},$$

which upper-bounds total expansions needed to reach all those solutions under the current policy. Optimizing this loss therefore corresponds to optimizing a provable upper bound on search effort.

A difficulty is that common policy parameterizations in modern AI, notably deep neural networks, render the loss highly nonconvex, complicating both theory and practice. The central contribution of [5] is to replace neural network policies with *parameterized context models* drawn from the online compression literature [14,15]. Contexts are organized into mutex sets, and their local action distributions are combined via product-of-experts mixing. Under a log-softmax parameterization, the authors show that the LTS loss is convex in the parameters, enabling standard convex optimization methods and regret-style guarantees in an online learning setting over found solution trajectories.

The paper then demonstrates empirically that this structured, convexly optimizable policy class can be competitive with and sometimes substantially outperform neural network-based variants on several benchmarks, including Sokoban (Boxoban), The Witness, and the 24-sliding-tile puzzle. They further report that LTS with context models can learn a satisficing policy for Rubik’s Cube that yields solutions within a few hundred expansions [5]. Importantly for practical adoption, the authors provide a reference implementation in Racket, along with instructions for reproducing the experimental results and a server–worker setup for parallel runs [6]. From the standpoint of Levin’s emphasis on constant overheads, this systems contribution is not incidental: universal search-style ideas often fail or succeed based on implementation overhead and engineering details.

### 4.3. Rerooting LTS: $\sqrt{\mathrm{LTS}}$ and Decomposition-Based Competitiveness

A limitation of root-based best-first search is structural: a node can only be reached after its ancestors have been visited. When useful side information appears only after reaching certain nodes, this constraint can be costly. Orseau, Hutter, and Lelis address this issue by introducing $\sqrt{\mathrm{LTS}}$ (pronounced root-LTS), which implicitly starts an LTS search rooted at every visited node and shares search effort across these rooted searches according to rerooting weights assigned by a rerooter [7].

Technically, the paper develops a refined self-counting cost function $\lambda_{\pi }$ (slenderness-based) to tighten counting arguments relative to the classic d/π cost, and it shows how to compose self-counting cost functions. This composition provides an implicit time-sharing scheme among many rooted searches, implemented within a single best-first enumeration. The main guarantee bounds the number of node visits to a target in terms of a chosen subtask decomposition along an ancestor chain and a factor that depends on rerooting-weight uncertainty. At the level of the arXiv abstract, the headline claim is that if LTS takes time *T*, then in a best case with *q* rerooting points, $\sqrt{\mathrm{LTS}}$ can take time on the order of

$$O\;\left(q\;\sqrt[{q}]{T}\right),$$

with robustness transformations proposed to avoid vacuous bounds when the number of “clues” becomes large or misleading [7].

From a universal search perspective, $\sqrt{\mathrm{LTS}}$ can be viewed as a second-order universalization. Rather than allocating effort only over trajectories from a fixed root, the algorithm allocates effort over a growing set of subsearches rooted at visited nodes. Rerooting weights function as a prior over these emerging candidates, and the self-counting composition of costs functions as the scheduling mechanism. The rerooter thus plays a role analogous to a learned or hand-crafted meta-prior over which encountered states should receive concentrated subsequent search.

## 5. Synthesis: What “Universality” Means Here

Across refs. [2,3,5,7], “universal search” is less a single algorithm than a design pattern that can be instantiated at multiple levels. Classically, the candidate set consists of programs. In policy-guided search, the candidate set consists of trajectories, with the policy playing the role of a prior. In rerooted search, the candidate set consists of implicit subsearches or decompositions, with rerooting weights playing the role of a dynamically revealed prior.

In each case, the guarantee is meaningful only relative to the representation. Universal search is optimal in its reference class, but the constant overhead depends on how candidates are encoded and how verification is performed. The more-recent studies make these trade-offs explicit. State cuts and policy mixing in [3] control redundant exploration and prevent brittle failure under miscalibration. Context models in [5] constrain the policy class so that the search bound can be optimized with convex methods, yielding both stability and theoretical learning guarantees that neural networks do not generally provide. Rerooting in [7] expands the class of “candidates” from trajectories to decompositions, enabling speedups when side information marks useful intermediate states, while simultaneously introducing new overhead-management challenges that are addressed via weight transformations.

Solomonoff induction remains a conceptual north star rather than a blueprint. Its universal mixture is defined over all computable hypotheses, but it is incomputable in general. The reviewed search methods inherit the same mixture-and-competition structure while restricting attention to action-centric candidates and to policy classes that can be learned with realistic compute. The context-model approach, in particular, suggests a pragmatic route for importing predictive/compressive structure into search priors while retaining optimization guarantees [5].

## 6. Applications to AI and Reinforcement Learning

The immediate AI interpretation of policy-guided search is that it offers a principled interface between learning and combinatorial exploration. Reinforcement learning can provide a policy that assigns probability mass to promising actions. Search then corrects policy errors by systematic exploration, and its guarantees are expressed directly in terms of the policy probabilities. This differs from traditional heuristic search where side information is typically a scalar heuristic estimate and guarantees are tied to admissibility or suboptimality. Here, the guarantee is about *effort to find any solution*, governed by probability mass on successful trajectories.

Orseau et al. emphasize a fundamental trade-off between enumeration and sampling [3]. In a needle-in-a-haystack regime, LevinTS is robust because it systematically explores trajectories in increasing $d_{0}/\pi$ cost, ensuring that no moderately probable short candidate is neglected. In a many-solution regime, sampling can exploit cumulative probability mass and achieve exponential improvements when there are many distinct solutions, and LubyTS provides a universal restart strategy that adapts to unknown solution depth distributions [4].

The learning-centric view becomes sharper in the context-model formulation [5]. Rather than training policies solely to act well (e.g., maximizing reward) or to imitate expert trajectories (e.g., minimizing cross-entropy), the policy is trained to reduce a bound on *search expansions*. This yields an objective aligned with planning performance and, through convexity, admits a kind of learning theory rarely available in neural approaches. The released implementation further suggests that, in this setting, systems considerations and overhead management are central: parallelism, caching, and stable numerics matter for turning a universal search-style guarantee into an actual speedup [6].

Finally, rerooting introduces a formal mechanism for subtask discovery [7]. In reinforcement learning terms, rerooting points resemble landmarks, bottlenecks, or shaping events that indicate progress. The novelty is that $\sqrt{\mathrm{LTS}}$ seeks efficiency guarantees depending on rerooting weights, rather than purely asymptotic convergence guarantees in stochastic settings. This opens a path toward principled use of intermediate signals in deterministic planning without sacrificing the correctness-by-search ethos. It is important to observe that these methods have demonstrated strong performance for structured deterministic puzzles such as Sokoban, sliding-tile problems, and these successes support the proposed “universal search as a design pattern” viewpoint.

## 7. Open Problems and Research Directions

A natural next step is to learn rerooters rather than hand-design them. The rerooter determines how search effort is distributed among implicit subsearches rooted at visited nodes [7]. One can ask whether there exists a loss analogous to the LTS loss for rerooting weights, whether rerooters can be learned from solved instances and solution trajectories, and whether one can obtain regret or convergence guarantees in training loops that resemble realistic bootstrap procedures.

A closely related direction is the joint learning of policy and decomposition. Context models provide convex learning for policies under an LTS-derived loss [5], while rerooting provides a decomposition mechanism that can yield qualitative speedups when side information is informative [7]. Jointly learning both may be crucial for performance, but it complicates theory: the appropriate comparator class for regret could be fixed policies, fixed decompositions, the best decomposition for a given policy, or the best pair. Clarifying what is achievable here would sharpen the sense in which these methods approximate universal search principles.

It is interesting to compare the Levin-style universal search with the heuristic Monte Carlo Tree Search. The two might be seen as complementary. The Levin universal search faces the scalability problem when the search universe is too large. Consider the game GO, which has about $10^{470}$ configurations compared to Sokoban with $10^{15}$ configurations only. The Monte Carlo Tree Search provides a heuristic solution of larger games such as GO. Being a PSPACE-hard problem (when the dimensionality of the game *n* is unbounded), it is unlikely that GO has simple solutions in general to be found by Levin search.

A broader and arguably deeper direction is to connect prediction-centric universality to action-centric search guarantees. Solomonoff induction provides a universal mixture for prediction across computable measures up to constants [8,9,11]. Policy-guided search provides universal-style guarantees relative to a policy and, in the rerooted case, relative to a rerooter. A tighter bridge would integrate these: predictive models would produce policies and rerooters, and one would track how predictive uncertainty translates into search-time guarantees. Even partial results could clarify when universal priors genuinely help in planning and when they merely introduce overhead.

## 8. Conclusions

Levin’s universal search provides a compelling ideal: a single meta-procedure competitive with the best solver in a broad class, with performance limited primarily by prior weights, verification cost, and overhead [2]. Solomonoff induction provides an analogous ideal for prediction by mixing over all computable hypotheses [8,9]. The recent policy-guided search line can be read as a careful specialization of universal search principles to deterministic planning. Trajectories replace programs, policies replace coding priors, and goal tests replace witness verification. Within this framing, LevinTS/LubyTS establish the core guarantee forms for enumeration and sampling [3]. LTS with context models shows how to learn search-effective policies with convex optimization by importing compression-inspired structure, and it provides a reference implementation that emphasizes the practical role of overhead [5,6]. Finally, rerooted $\sqrt{\mathrm{LTS}}$ lifts the universality pattern to decompositions by allocating effort across many implicit rerooted searches, yielding competitiveness with the best subtask decomposition under uncertainty [7]. A unifying lesson is that universality is not free: the practical impact depends on how well we design priors (policies), representations (contexts), and discovery mechanisms (rerooters) to control overhead while preserving meaningful guarantees.

## Data Availability

No new data were created or analyzed in this study.
